# Prognosis, characteristics, and provision of care for patients with the unspecified heart failure electronic health record phenotype: a population-based linked cohort study of 95262 individuals

**DOI:** 10.1016/j.eclinm.2023.102164

**Published:** 2023-08-25

**Authors:** Yoko M. Nakao, Kazuhiro Nakao, Ramesh Nadarajah, Amitava Banerjee, Gregg C. Fonarow, Mark C. Petrie, Kazem Rahimi, Jianhua Wu, Chris P. Gale

**Affiliations:** a Leeds Institute of Cardiovascular and Metabolic Medicine, University of Leeds, Leeds, UK; b Leeds Institute for Data Analytics, University of Leeds, Leeds, UK; c Department of Pharmacoepidemiology, Graduate School of Medicine and Public Health, Kyoto University, Kyoto, Japan; d Department of Cardiovascular Medicine, National Cerebral and Cardiovascular Center, Suita, Japan; e Department of Cardiology, Leeds Teaching Hospitals NHS Trust, Leeds, UK; f Institute of Health Informatics, University College London, London, UK; g Department of Cardiology, Barts Health NHS Trust, London, UK; h Division of Cardiology, Department of Medicine, University of California at Los Angeles, Los Angeles, USA; i Institute of Cardiovascular and Medical Sciences, University of Glasgow, Glasgow, UK; j Nuffield Department of Women's and Reproductive Health, University of Oxford, Oxford, UK; k National Institute of Health Research Oxford Biomedical Research Centre, Oxford University Hospitals NHS Foundation Trust, Oxford, UK; l Deep Medicine, Oxford Martin School, University of Oxford, Oxford, UK; m Oxford University Hospitals NHS Foundation Trust, Oxford, UK; n Wolfson Institute of Population Health, Queen Mary University of London, London, UK

**Keywords:** Heart failure, Electronic health records, Care quality, Outcome

## Abstract

**Background:**

Whether the accuracy of the phenotype ascribed to patients in electronic health records (EHRs) is associated with variation in prognosis and care provision is unknown. We investigated this for heart failure (HF, characterised as HF with preserved ejection fraction [HFpEF], HF with reduced ejection fraction [HFrEF] and unspecified HF).

**Methods:**

We included individuals aged 16 years and older with a new diagnosis of HF between January 2, 1998 and February 28, 2022 from linked primary and secondary care records in the Clinical Practice Research Datalink in England. We investigated the provision of guideline-recommended diagnostic investigations and pharmacological treatments. The primary outcome was a composite of HF hospitalisation or all-cause death, and secondary outcomes were time to HF hospitalisation, all-cause death and death from cardiovascular causes. We used Kaplan–Meier curves and log rank tests to compare survival across HF phenotypes and adjusted for potential confounders in Cox proportional hazards regression analyses.

**Findings:**

Of a cohort of 95,262 individuals, 1271 (1.3%) were recorded as having HFpEF, 10,793 (11.3%) as HFrEF and 83,198 (87.3%) as unspecified HF. Individuals recorded as unspecified HF were older with a higher prevalence of dementia. Unspecified HF, compared to patients with a recorded HF phenotype, were less likely to receive specialist assessment, echocardiography or natriuretic peptide testing in the peri-diagnostic period, or receive angiotensin-converting enzyme inhibitors, beta blockers or mineralocorticoid receptor antagonists up to 12 months after diagnosis (risk ratios compared to HFrEF, 0.64, 95% CI 0.63–0.64; 0.59, 0.58–0.60; 0.57, 0.55–0.59; respectively) and had significantly worse outcomes (adjusted hazard ratios compared to HFrEF, HF hospitalisation and death 1.66, 95% CI 1.59–1.74; all-cause mortality 2.00, 1.90–2.10; cardiovascular death 1.77, 1.65–1.90).

**Interpretation:**

Our findings suggested that absence of specification of HF phenotype in routine EHRs is inversely associated with clinical investigations, treatments and survival, representing an actionable target to mitigate prognostic and health resource burden.

**Funding:**

Japan Research Foundation for Healthy Aging and 10.13039/501100000274British Heart Foundation.

**Research in context**

**Evidence before this study**

We searched Medline and Embase for reports published in English from inception to January 30, 2023 with a combination of keywords and subject headings related to heart failure (HF), prognosis, care quality, and phenotypes (reduced ejection fraction [HFrEF], preserved ejection fraction [HFpEF]. We also reviewed reference lists of selected reports. Previous research into community-dwelling individuals with HF have reported shortfalls in provision of care across multiple geographies, including the UK, but lack detailed information about associations with prognosis. A UK-based study found that the recording of HF phenotype in primary care electronic health records (EHRs) was poor but did not investigate variation by patient characteristics or diagnostic setting. We found no study that studied whether the recording of HF phenotype in EHRs was associated with prognosis or extent of care provision.

**Added value of this study**

Our study provides novel information on the paucity of recording of HF phenotype in primary care records in a large cohort of patients with HF in England, and its association with prognosis and care provision. Nine in ten patients with HF did not have their phenotype coded, and this particularly affected older people who had been diagnosed in hospital. An absence of phenotype-specific coding was associated with fewer guideline-recommended investigations and less use of disease-modifying pharmacotherapies. Individuals with unspecified HF, compared to those where HFrEF or HFpEF was coded, were twice as likely to die, even after accounting for their age, demography and comorbidity.

**Implications of all the available evidence**

The recording of HF phenotype is remarkably poor in primary care records and this is associated with different care and worse outcomes. HF is more common in England than the four most common causes of cancer combined, and has a worse prognosis than some cancer types, yet imprecise recording of phenotype may lead to unwarranted and unacceptable variation in care. The unspecified HF EHR phenotype represents an actionable target to improve the care pathway and disease trajectory for patients with HF. Insufficient phenotype recording in EHRs is also present for other common chronic diseases, such as chronic kidney disease, and whether our findings translate to other diseases merits further consideration.

## Introduction

Electronic health records (EHRs) have become ubiquitous across clinical practice in primary and secondary care and inform patient decision making and policy. Recording of diagnoses in EHRs is often considered a by-product of care, rather than as contributory to the care process itself. Whether the accuracy of the phenotype ascribed to patients in EHRs is associated with variation in prognosis has not been investigated.

Heart failure (HF) is a common condition with a poor prognosis, and has traditionally been divided into distinct phenotypes based on the measurement of left ventricular ejection fraction (LVEF)—HF with reduced ejection fraction (HFrEF, <40%), HF with mildly reduced ejection fraction (HFmrEF, 41–49%) and HF with preserved ejection fraction (HFpEF, >50%). Pharmacotherapy with angiotensin-converting enzyme inhibitors (ACEIs)/angiotensin receptor-neprilysin inhibitors (ARNIs), beta-blockers and mineralocorticoid receptor antagonists (MRAs) improves outcomes in patients with HFrEF, and receive class I recommendations in international guidelines, but this does not extend to patients with HFpEF.^1^^,^^2^ Investigations and stepwise initiation of pharmacotherapies for patients with HF mostly occurs in a non-inpatient setting,^3^ and in the UK general practitioners remain responsible for medication prescriptions. It is increasingly recognised that there is a shortfall in the recording of HF phenotypes in UK primary care EHRs,^4^ but previous reports have not investigated whether this may be associated with differences in prognosis or care provision.^3^^,^^5^, ^6^, ^7^, ^8^

To address this knowledge gap, we used a nationwide longitudinal database of linked primary and secondary care records from a representative sample of the English population to assess prognosis (all-cause and cardiovascular specific death) and provision of care for patients with a recorded HF phenotype compared with those where HF phenotype was not otherwise specified (unspecified).

## Methods

### Data source

We used electronic health records from the Clinical Practice Research Datalink (CPRD). The CPRD database contains anonymised patient data from approximately 7% of the UK population and is broadly representative in terms of age, sex, and ethnicity.^9^ CPRD is one of the largest databases of longitudinal medical records from primary care in the world and has been validated for epidemiological research for a broad range of conditions. Primary care records from CPRD were linked to secondary care admission records from Hospital Episodes Statistics Admitted Patient Care (HES-APC) data. Linkage was available for a subset of English practices from Jan 2, 1998, covering approximately 50% of all CPRD records. Previous research has demonstrated the representativeness of patients eligible for linkage in terms of age, gender and geography.^10^ This study based in part on data from the CPRD which has ethics approval from the Health Research Authority to support research using anonymised patient data. Scientific approval for this study was given by the CPRD Independent Scientific Advisory Committee (ISAC) (ref no: 21_000324).

### Study population

Patients were individuals aged 16 years and older of both sexes, contributing to data between January 2, 1998, and February 28, 2022. Patients were eligible for inclusion if their record was labelled as acceptable by CPRD quality control,^9^ approved for CPRD and HES-APC linkage, and if they were registered with their general practice for at least 12 months.

We excluded all individuals who had a diagnosis of HF before the study start date (January 2, 1998, in primary care records and secondary care records), or within the first 12 months of registration with their general practice. We also excluded one patient who was known to have died but for whom the date of death was missing.

### Heart failure diagnosis and phenotype

We defined incident HF as the first record of HF in primary care (Read code) or hospital admission records (International Classification of Diseases, tenth revision [ICD-10]) from any diagnostic position using a comprehensive set of diagnostic codes (Appendix Table 1).^11^^,^^12^ HF was stratified as HFpEF, HFrEF, or unspecified HF by the first diagnostic codes (Appendix Table 2). For patients with unspecified HF by the first code, if there was a HFpEF or HFrEF code within 180 days, it was reclassified as HFpEF or HFrEF. If there were both HFpEF and HFrEF codes on the same day, they were classified as HFrEF. We could not identify HFmrEF using the available diagnostic codes.

### Patient characteristics

We used the Index of Multiple Deprivation (IMD) 2019 quintile to describe socioeconomic status.^13^ To calculate body mass index (BMI, kg/m^2^), we extracted the most recent measurement of body weight within 1 year of a diagnosis of HF. If the weight was measured beyond 1 year of the HF diagnosis, we categorized it as unrecorded for BMI. To describe comorbidities, we selected five cardiovascular comorbidities (atrial fibrillation, hypertension, ischaemic heart disease, valvular heart disease and ischaemic stroke) and 11 non-cardiovascular comorbidities (anaemia, cancer, chronic kidney disease, chronic obstructive pulmonary disease, dementia, depression, diabetes, dyslipidaemia, gout, sleep apnoea syndrome and thyroid disease). For each condition, we report prevalence as the percentage of patients with a diagnosis recorded in their primary care or hospital discharge record, before their first diagnosis of HF. Diagnosis code lists for the extraction of each condition were adapted from the CALIBER code repository. Frailty was ascertained on the date of HF diagnosis using the electronic frailty index (eFI), which includes 36 equally weighted deficit variables, based on Read codes (Appendix).

### Care provision

The setting in which HF was first diagnosed was categorised as either inpatient or outpatient. Outpatient diagnoses refer to diagnoses first recorded in primary care with no prior HF hospitalisation and are likely to reflect both outpatient consultations by specialists and direct diagnoses by general practitioners.

We studied the provision of European Society of Cardiology (ESC) guideline-recommended diagnostic investigations including echocardiogram, 12-lead electrocardiogram (ECG), plasma natriuretic peptides (B-type natriuretic peptide [BNP] or N-terminal-pro-BNP), chest x-ray and other blood tests (full blood count, urea and electrolytes, thyroid function, fasting glucose and HbA1c, lipids, and iron status) within 3 months of incident HF diagnosis (Appendix Fig. 2).^1^ Diagnostic tests were considered individually and as a composite of any of the five diagnostic investigations. We also assessed a cardiology specialist assessment within 3 months of incident HF diagnosis.

Drug treatment patterns were investigated for the main treatment classes indicated in the management of HF^1^: ACEIs or angiotensin receptor blockers (ARBs), ARNIs; beta blockers, diuretics, MRAs and sodium-glucose co-transporter-2 inhibitor (SGLT2i) (Appendix Table 3). For each of the drug classes, we report treatment initiation as the proportion of eligible patients who received at least one prescription in the first 3, 6 and 12 months following their HF diagnosis.

### Outcomes

The primary outcome was a composite outcome of time to first hospitalisation for HF or all-cause death. We obtained the date and cause of death from the Office for National Statistics mortality data. Secondary outcomes were time to first hospitalisation for HF, all-cause death and death from cardiovascular causes.

### Statistical analysis

Baseline characteristics are presented as frequencies (%) for categorical data or means and standard deviations (SD) for continuous data.

There were missing data for ethnicity, IMD, BMI and smoking status. We considered multiple imputation inappropriate as comparison of the characteristics of people with and without missing data suggested the data were not missing at random.^12^ Patients with missing ethnicity data were default imputed as white,^14^ and patients with missing smoking data as non-smokers.^15^ IMD data were missing for 16 (0.06%) of HFrEF and 64 (0.05%) of unspecified HF. We excluded patients with missing IMD data from multivariate analyses. Unrecorded BMI data were represented by an additional missing category.

To examine changes in care provision over time and by subgroups, we used a Poisson model with robust error variance and report risk ratios (RRs) alongside corresponding 95% confidence intervals (CIs). All models were adjusted for year of diagnosis, age, sex and socioeconomic status (IMD quintile). Selected graphical representations were smoothed using local polynomial regression.^16^

We used Kaplan–Meier curves and log rank tests to compare the primary and secondary outcomes across HF phenotypes. Cox proportional hazards regression analyses assessed the overall effect of HF phenotypes on outcomes adjusted for potential confounders. We assessed the consistency of the main results by sex (men, women), socioeconomic status (IMD quintile) and diagnostic setting (outpatient, inpatient). We conducted the following sensitivity analyses: 1) the missing values for ethnicity and smoking were included as a separate category, 2) the cohort was restricted to only HF diagnoses from 2010 to take into account iterations of HF guidelines, and 3) differences in prescription of RAAS blockade and beta blockers were adjusted for to assess how this affected outcomes.

Study findings are reported in accordance with the Reporting of studies Conducted using Observational Routinely-collected health Data (RECORD) recommendations^17^ and CODE-EHR framework.^18^ We used STATA, version 17.0 to perform statistical analysis with statistical significance was set at p < 0.05.

### Role of the funding source

The funders of the study had no role in study design, data collection, data analysis, data interpretation, or writing the report. YMN, KN, RN, JW and CPG had full access to all data in the study. All authors accept responsibility to submit for publication.

## Results

**Fig. 1:**
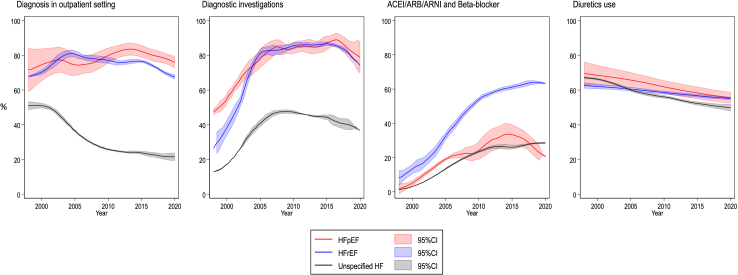
**Temporal trends in diagnostic care setting and pharmacotherapy prescription by recorded HF phenotype**. Results from 1998 to 2020 are presented as fitted local polynomial regression over yearly averages and 95% confidence intervals (shade). ACEI: Angiotensin-converting-enzyme inhibitor; ARB: Angiotensin receptor blocker; ARNI: Angiotensin receptor-neprilysin inhibitor; CI: confidence interval; HF: Heart failure; HFpEF: Heart failure with preserved ejection fraction; HFrEF: Heart failure with reduced ejection fraction.

95,262 patients with newly diagnosed HF between 1998 and 2022 were included in the study (Appendix Fig. 1). At the time of HF diagnosis, the mean age was 76.7 years (SD 12.1; interquartile range 70–85), 46,847 (49%) patients were women and 63,899 (67%) patients had three or more of the included comorbidities. For the majority of patients, HF phenotype was unspecified (83,198, 87%). Compared to patients with a recorded HF phenotype, patients with unspecified HF were older, three-fold more likely to have dementia at time of HF diagnosis, and less likely to have data recorded for BMI, ethnicity and smoking status (Table 1). Two-thirds of patients with unspecified HF were originally diagnosed during a hospital admission, whereas four-fifths of patients with HFrEF or HFpEF had been diagnosed in an outpatient setting (Table 2; Fig. 1). For the majority of unspecified HF cases originally diagnosed in an inpatient setting, HF was not the primary reason for admission (76%; Appendix Table 4).

**Table 1:** Baseline characteristics of people with heart failure phenotypes.

	HFrEF	HFpEF	Unspecified HF
	n = 10,793	n = 1271	n = 83,198
Age (years)	69.9 (13.2)	74.4 (11.2)	77.6 (11.7)
Sex			
Men	7087 (65.7%)	580 (45.6%)	40,748 (49.0%)
Women	3706 (34.3%)	691 (54.4%)	42,450 (51.0%)
Ethnicity			
White	9925 (92.0%)	1146 (90.2%)	73,085 (87.8%)
Others	465 (4.3%)	87 (6.8%)	2807 (3.4%)
Missing	403 (3.7%)	38 (3.0%)	7306 (8.8%)
Socioeconomic status quintile			
1 (least deprived)	1993 (18.5%)	208 (16.4%)	14,320 (17.2%)
2	2077 (19.2%)	236 (18.6%)	16,340 (19.6%)
3	2467 (22.9%)	284 (22.3%)	18,258 (21.9%)
4	2230 (20.7%)	274 (21.6%)	18,152 (21.8%)
5 (most deprived)	2020 (18.7%)	269 (21.2%)	16,079 (19.3%)
Missing	6 (0.1%)	0 (0.0%)	49 (0.1%)
Smoking			
Ever	6834 (63.3%)	748 (58.9%)	44,064 (53.0%)
No	1901 (17.6%)	281 (22.1%)	11,135 (13.4%)
Missing	2058 (19.1%)	242 (19.0%)	27,999 (33.7%)
BMI (kg/m^2^)	28.9 (6.2)	29.8 (6.4)	28.3 (6.8)
BMI category			
Underweight	90 (0.8%)	12 (0.9%)	1189 (1.4%)
Normal	1426 (13.2%)	161 (12.7%)	9175 (11.0%)
Overweight	2054 (19.0%)	226 (17.8%)	10,240 (12.3%)
Obesity class I	1314 (12.2%)	181 (14.2%)	6170 (7.4%)
Obesity class II/III	798 (7.4%)	136 (10.7%)	4497 (5.4%)
Missing	5111 (47.4%)	555 (43.7%)	51,927 (62.4%)
Cardiovascular comorbidities			
Atrial fibrillation	3666 (34.0%)	364 (28.6%)	30,291 (36.4%)
Hypertension	6825 (63.2%)	947 (74.5%)	53,967 (64.9%)
Ischaemic heart disease	5615 (52.0%)	531 (41.8%)	40,154 (48.3%)
Stroke	1321 (12.2%)	181 (14.2%)	10,645 (17.6%)
Valvular heart disease	2107 (19.5%)	240 (18.9%)	13,269 (15.9%)
Non-cardiovascular comorbidities			
Anaemia	2068 (19.2%)	316 (24.9%)	21,427 (25.8%)
Cancer	1531 (14.2%)	201 (15.8%)	14,320 (17.2%)
Chronic kidney disease	1439 (13.3%)	224 (17.6%)	13,514 (16.2%)
COPD	1617 (15.0%)	215 (16.9%)	17,075 (20.5%)
Dementia	189 (1.8%)	24 (1.9%)	5065 (613%)
Depression	2241 (20.8%)	289 (22.7%)	15,660 (18.8%)
Diabetes	2436 (22.6%)	309 (24.3%)	19,060 (22.9%)
Dyslipidaemia	3736 (34.6%)	463 (36.4%)	20,628 (24.8%)
Gout	1071 (9.9%)	121 (9.5%)	7574 (9.1%)
Sleep apnoea syndrome	194 (1.8%)	29 (2.3%)	992 (1.2%)
Thyroid disease	995 (9.2%)	173 (13.3%)	9605 (11.5%)
Three or more comorbidities	6889 (63.8%)	850 (66.9%)	54,941 (66.0%)
Frailty			
Fit	2935 (27.2%)	193 (15.2%)	18,645 (22.4%)
Mild frailty	4778 (44.3%)	577 (45.4%)	34,960 (42.0%)
Moderate frailty	2371 (22.0%)	374 (29.4%)	21,916 (26.3%)
Severe frailty	709 (6.6%)	127 (10.0%)	7677 (9.2%)

Mean (standard deviation) or number (%).

BMI: Body mass index; HF: Heart failure; COPD: Chronic obstructive pulmonary disease HFpEF: Heart failure with preserved ejection fraction; HFrEF: Heart failure with reduced ejection fraction.

**Table 2:** Investigations and treatments by heart failure phenotypes.

	HFrEF	HFpEF	Unspecified HF	Risk ratio (95% CI) HFrEF as reference	
	n = 10,793	n = 1271	n = 83,198	HFpEF	Unspecified HF
**Diagnosis care setting**					
Outpatient	8263 (76.6%)	1009 (79.4%)	30,129 (36.2%)	1.05 (1.02–1.09)	0.40 (0.39–0.41)
Inpatient (HF primary cause)	973 (9.0%)	96 (7.6%)	12,831 (15.4%)	0.80 (0.65–0.98)	1.58 (1.48–1.68)
Inpatient (HF secondary cause)	1557 (14.4%)	166 (13.1%)	40,238 (48.4%)	0.91 (0.78–1.05)	3.87 (3.69–4.05)
**Diagnostic investigation**					
Echocardiogram	7536 (69.8%)	892 (70.2%)	13,832 (16.6%)	1.05 (1.01–1.09)	0.33 (0.32–0.33)
ECG	3767 (34.9%)	443 (34.9%)	13,183 (15.8%)	1.05 (0.97–1.14)	0.59 (0.57–0.61)
NP test	956 (8.9%)	155 (12.2%)	3697 (4.4%)	1.32 (1.13–1.54)	0.73 (0.68–0.79)
Chest x-ray	2343 (21.7%)	277 (21.8%)	13,079 (15.7%)	1.03 (0.92–1.14)	0.84 (0.81–0.88)
Other blood tests					
Full blood count	1359 (12.6%)	152 (12.0%)	10,651 (12.8%)	0.89 (0.76–1.03)	0.99 (0.94–1.05)
Urea and/or electrolytes	3220 (29.8%)	412 (32.4%)	22,265 (26.8%)	1.05 (0.97–1.15)	1.01 (0.98–1.05)
Thyroid function	2612 (24.2%)	275 (21.6%)	14,272 (17.2%)	0.85 (0.76–0.95)	0.82 (0.79–0.85)
Fasting glucose/HbA1c	2906 (26.9%)	335 (26.4%)	16,458 (19.8%)	0.98 (0.89–1.08)	0.92 (0.89–0.95)
Lipids	2709 (25.1%)	311 (24.5%)	12,904 (15.5%)	1.04 (0.94–1.15)	0.83 (0.80–0.87)
Iron status	796 (7.4%)	113 (8.9%)	4910 (5.9%)	1.11 (0.93–1.34)	1.05 (0.97–1.13)
All blood tests above	35 (0.3%)	5 (0.4%)	266 (0.3%)	1.14 (0.45–2.91)	1.26 (0.88–1.82)
At least 1 diagnostic investigation	8668 (80.3%)	1038 (81.7%)	29,294 (35.2%)	1.05 (1.02–1.08)	0.55 (0.55–0.56)
Specialist assessment	1313 (12.2%)	160 (12.6%)	3481 (4.2%)	1.12 (0.96–1.31)	0.51 (0.48–0.54)
**Treatment initiation**					
**Within 3 months**					
ACEI	7512 (69.6%)	625 (49.2%)	33,042 (39.7%)	0.76 (0.72–0.81)	0.63 (0.62–0.64)
ARB	1506 (14.0%)	232 (18.3%)	6946 (8.3%)	1.30 (1.15–1.47)	0.76 (0.71–0.80)
ARNI	23 (0.2%)	0 (0.0%)	14 (<1%)	–	–
Beta blocker	5974 (55.4%)	414 (32.6%)	17,275 (20.8%)	0.65 (0.60–0.70)	0.60 (0.59–0.61)
Diuretics	6284 (58.2%)	784 (61.7%)	49,543 (59.5%)	1.02 (0.98–1.07)	0.91 (0.90–0.93)
MRA	2283 (21.2%)	147 (11.6%)	7776 (9.3%)	0.59 (0.50–0.69)	0.58 (0.56–0.61)
SGLT2i	30 (0.3%)	2 (0.2%)	59 (0.1%)	0.90 (0.21–3.98)	0.66 (0.42–1.06)
**Within 6 months**					
ACEI	7914 (73.3%)	667 (52.5%)	35,301 (42.4%)	0.77 (0.73–0.82)	0.63 (0.62–0.64)
ARB	1815 (16.8%)	256 (20.1%)	7970 (9.6%)	1.20 (1.07–1.35)	0.71 (0.67–0.74)
ARNI	44 (0.4%)	0 (0.0%)	20 (<1%)	–	–
Beta blocker	6505 (60.3%)	458 (36.0%)	18,751 (22.5%)	0.66 (0.61–0.71)	0.59 (0.58–0.60)
Diuretics	6656 (61.7%)	841 (66.2%)	52,535 (63.1%)	1.04 (0.99–1.08)	0.91 (0.90–0.93)
MRA	2693 (25.0%)	181 (14.2%)	9216 (11.1%)	0.61 (0.53–0.70)	0.57 (0.55–0.60)
SGLT2i	45 (0.4%)	3 (0.2%)	76 (0.1%)	0.83 (0.25–2.81)	0.55 (0.37–0.81)
**Within 12 months**					
ACEI	8177 (75.8%)	699 (55.0%)	37,125 (44.6%)	0.78 (0.74–0.82)	0.64 (0.63–0.64)
ARB	2138 (19.8%)	282 (22.2%)	9018 (10.8%)	1.13 (1.02–1.26)	0.67 (0.64–0.70)
ARNI	80 (0.7%)	0 (0.0%)	44 (0.1%)	–	–
Beta blocker	6908 (64.0%)	483 (38.0%)	20,129 (24.2%)	0.66 (0.61–0.70)	0.59 (0.58–0.60)
Diuretics	6982 (64.7%)	881 (69.3%)	54,257 (65.2%)	1.04 (1.00–1.08)	0.90 (0.89–0.92)
MRA	3105 (28.8%)	203 (16.0%)	10,679 (12.8%)	0.60 (0.52–0.68)	0.57 (0.55–0.59)
SGLT2i	59 (0.5%)	3 (0.2%)	93 (0.1%)	0.60 (0.18–1.97)	0.49 (0.36–0.70)

Risk ratios and 95% confidence intervals comparing heart failure with preserved ejection fraction (HFpEF) and unspecified heart failure to heart failure with reduced ejection fraction (HFrEF) (reference), adjusting for year of diagnosis, age, sex and socioeconomic status.

ACEI: Angiotensin-converting-enzyme inhibitor; ARB: Angiotensin receptor blocker; ARNI: Angiotensin receptor-neprilysin inhibitor; CI: Confidence interval; ECG: Electrocardiogram; HF: Heart failure; HFpEF: Heart failure with preserved ejection fraction; HFrEF: Heart failure with reduced ejection fraction; MRA: Mineralocorticoid receptor antagonist; NP: Natriuretic peptide; SGLT2i: Sodium-glucose cotransporter-2 inhibitors.

Similar proportions of patients classified as HFrEF or HFpEF had a record of an echocardiogram, ECG, blood tests and chest x-ray (Table 2). However, patients with unspecified HF were less likely to have a record of a specialist assessment in the peri-diagnosis period, or have been recorded to have been investigated with an echocardiogram, ECG, natriuretic peptide test and chest x-ray (RR 0.33, 95% CI 0.32–0.33; 0.59, 0.57–0.61; 0.73 0.68–0.79; and 0.84, 0.81–0.88). There was a trend to declining provision of diagnostic investigations for patients with unspecified HF from 2010 onwards (Fig. 1).

Patients with unspecified HF were also subject to lower rates of initiation of guideline-recommended treatment in primary care over the first 12 months after HF diagnosis (Table 2). The majority were prescribed diuretics, with prescription rates numerically similar to patients classified with HFrEF and HFpEF (Table 2), but the rates of renin-angiotensin-aldosterone system (RAAS) blockade and beta blocker prescription were lower compared with individuals with HFrEF and this trend was consistent across the first 12 months after diagnosis (RR [95% CI] compared to HFrEF over 3, 6, and 12 months, for ACEI: 0.63 [0.62–0.64], 0.63 [0.62–0.64], 0.64 [0.63–0.64]; for beta blockers: 0.60 [0.59–0.61], 0.59 [0.58–0.61], and 0.59 [0.58–0.60]).

**Fig. 2:**
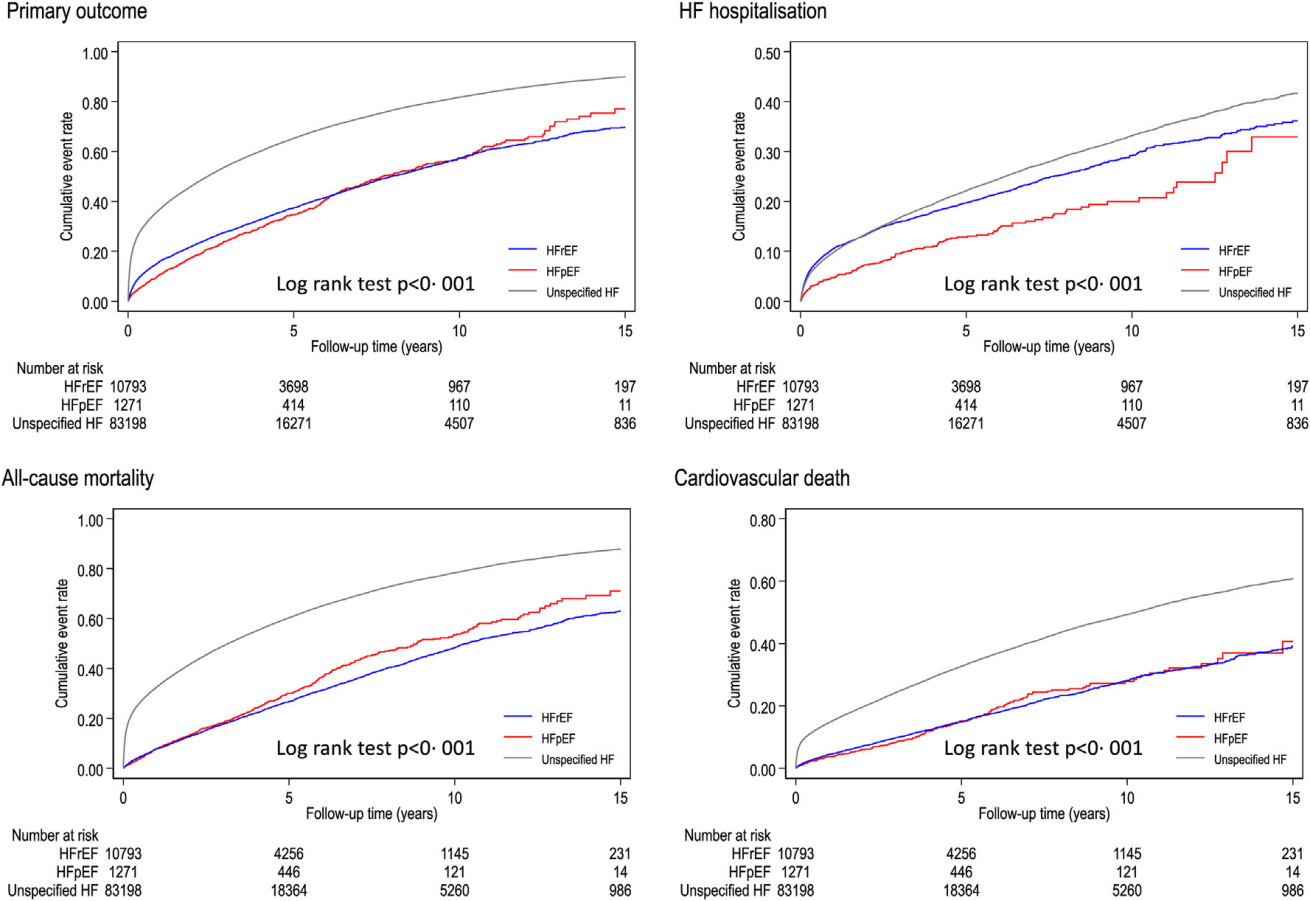
**Kaplan–Meier curves.** HF: Heart failure; HFpEF: Heart failure with preserved ejection fraction; HFrEF: Heart failure with reduced ejection fraction.

Patients with unspecified HF more frequently experienced adverse clinical outcomes during follow up compared with patients recorded as HFpEF or HFrEF (Fig. 2), with a crude incidence rate for the composite outcome (HF hospitalisation or all-cause death) almost three-fold higher (HFrEF 9.7 per 100-person year, 95% CI 9.4–9.9; HFpEF 9.2, 8.4–10.0; unspecified HF 24.0, 23.8–24.2, Table 3). Compared with patients with a HF phenotype, patients with unspecified HF had an increased risk of the composite outcome (adjusted HR compared to HFrEF, 1.71, 95% CI 1.66–1.76, p < 0.01) and were at higher risk of both death from any cause (2.09, 2.01–2.16, p < 0.01) and, specifically, cardiovascular death (1.79, 1.71–1.89, p < 0.01).

**Table 3:** Association between heart failure phenotypes and outcomes.

	Number of events	Crude incidence per 100 person-years (95% CI)	Adjusted HR (95% CI)	p value
**Primary outcome**				
HFrEF	4402	9.7 (9.4–9.9)	Reference	
HFpEF	480	9.2 (8.4–10.0)	0.83 (0.75–0.91)	<0.01
Unspecified HF	54,616	24.0 (23.8–24.2)	1.71 (1.66–1.76)	<0.01
**Secondary outcomes**				
HF hospitalisation				
HFrEF	2053	4.5 (4.3–4.7)	Reference	
HFpEF	149	2.8 (2.4–3.3)	0.57 (0.49–0.68)	<0.01
Unspecified HF	12,369	5.4 (5.3–5.5)	0.98 (0.93–1.03)	0.37
All-cause mortality				
HFrEF	3370	6.5 (6.3–6.8)	Reference	
HFpEF	423	7.6 (6.9–8.3)	0.99 (0.89–1.09)	0.80
Unspecified HF	51,149	20.4 (20.2–20.6)	2.09 (2.01–2.16)	<0.01
Cardiovascular death				
HFrEF	1728	3.4 (3.2–3.5)	Reference	
HFpEF	186	3.3 (2.9–3.8)	0.87 (0.74–1.01)	0.06
Unspecified HF	21,807	8.7 (8.6–8.8)	1.79 (1.71–1.89)	<0.01

Model was adjusted for age, sex, ethnicity, socioeconomic status, smoking, body mass index, atrial fibrillation, hypertension, ischaemic heart disease, stroke, valvular heart disease, anaemia, cancer, chronic kidney disease, chronic obstructive pulmonary disease, dementia, depression, diabetes, dyslipidaemia, gout, sleep apnoea syndrome and thyroid disease.

CI: Confidence interval; HF: Heart failure; HR: Hazard ratio; HFpEF: Heart failure with preserved ejection fraction; HFrEF: Heart failure with reduced ejection fraction.

On subgroup analysis we found that the record of provision of ECG or echocardiogram in the peri-diagnostic period, and initiation of beta blockers and RAAS blockers or MRAs up to 12 months after diagnosis, were lower for individuals with unspecified HF whether originally diagnosed as an inpatient and outpatient (Appendix Table 5). The magnitude of the shortfall in prescription of guideline-recommended treatment for individuals with unspecified HF, compared to those specified as HFpEF or HFrEF, was larger amongst patients who were diagnosed during an inpatient admission (RR compared to HFrEF at 12 months, for ACEI: outpatient 0.82, 95% CI 0.81–0.84; inpatient 0.57, 0.56–0.59; for beta blockers: outpatient 0.74, 0.72–0.76; inpatient 0.51, 0.49–0.52).

**Fig. 3:**
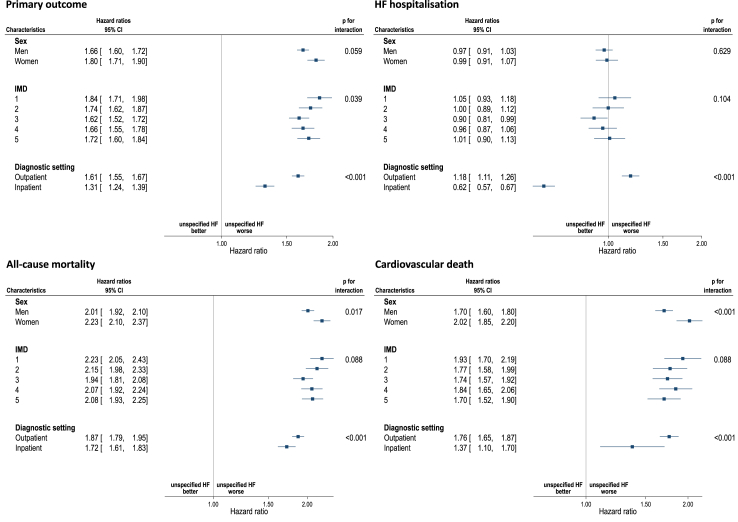
**Hazard ratio for unspecified HF compared to HFrEF by sex, socioeconomic status, and diagnosis care setting**. Model was adjusted for age, sex, ethnicity, socioeconomic status, smoking, body mass index, atrial fibrillation, hypertension, ischaemic heart disease, stroke, valvular heart disease, anaemia, cancer, chronic kidney disease, chronic obstructive pulmonary disease, dementia, depression, diabetes, dyslipidaemia, gout, sleep apnoea syndrome and thyroid disease. HF: Heart failure; HFrEF: Heart failure with reduced ejection fraction; IMD: Index of Multiple Deprivation.

The increased risk of adverse outcomes for patients with unspecified HF for the composite outcome, all-cause mortality and cardiovascular death was consistent across both sexes, in both the most affluent and deprived individuals, and whether diagnosis was originally made in an inpatient or outpatient setting (Fig. 3). The results were not altered in the sensitivity analyses where missing data for ethnicity and smoking were included as a separate category or where the cohort was restricted to HF diagnoses since 2010 (Appendix Tables 6 and 7); and worse ouctomes observed in the unspecified HF cohort persisted after adjustment for differences in prescription of neurohormonal blockade.

## Discussion

In this study of almost 100,000 patients with HF in England, we present novel findings regarding the paucity of HF phenotype recording in routine clinical records and the association with prognosis. For nine in ten patients with HF, their phenotype was not coded in structured medical record data, and this particularly affected older people who had been diagnosed in hospital and more commonly had dementia. The lack of specification of a HF phenotype was inversely associated with survival and the provision of guideline-recommended investigations and disease-modifying pharmacotherapies. Together these findings signal the missed opportunity to more precisely record patients’ HF phenotypes in community EHRs and thereby potentially improve their disease trajectories.

Previous reports have described the provision of guideline-recommended diagnostic investigations^3^ and pharmacological treatment^5^^,^^8^ for community-dwelling patients with HF in the UK. To the best of our knowledge our study is the first to consider the contribution of precise recording of HF phenotype in community EHRs. Detailed clinical coding may be seen as a research practice but we demonstrate that it has real clinical consequences–the unspecified HF EHR phenotype is associated with differences in management and worse outcomes for patients. Randomised controlled trials have demonstrated that sodium glucose cotransporter 2 inhibitors improve outcomes in individuals with HF,^19^ but imprecise coding may mask the eligibility of patients to receive these medications, leading to delays in prescription and failure to realise an opportunity to reduce downstream morbidity, mortality and health expenditure. Accurate coding is an important lever to improve care throughout the care pathway across diseases.

The failure to accurately record HF phenotypes in UK EHRs is of particular concern given the major public health challenge that HF represents. There are more cases of HF diagnosed each year in the UK NHS than the four most common causes of cancer combined (lung, breast, prostate and bowel),^11^ HF is the most common cause of unplanned hospital admission in older persons,^20^ and expenditure from HF is projected to rise disproportionately compared to expenditure on other major morbidities such myocardial infarction and cancer.^21^ It would be unacceptable to record a diagnosis of cancer without specifying the type of cancer and targeting treatments to that cancer type, yet we found this was common for HF even though it has a worse prognosis than each of bladder, prostate and breast cancer.^22^ Amongst patients with HF about half have HFpEF and half have HFrEF,^23^ so it is possible that approximately fourty thousand patients in our cohort had uncharacterised HFrEF, and were subject to inequity, with beta blocker and RAAS blocker prescription rates between two-to-three fold lower than their counterparts with recorded HFrEF.

Imprecision in HF phenotype recording may result from inadequate information exchange between hospitals and primary care. Previous reports have demonstrated that up to a third of HF diagnoses in the UK are recorded in hospital admissions without associated recording in primary care, and that these individuals are subject to worse outcomes.^7^ In our study, the vast majority of patients with HF originally diagnosed in hospital did not have a recorded HF phenotype in their community records within 6 months. These patients were older, more likely to have dementia and HF had not been the main reason for admission. This subcohort had poor care delivery and a particularly poor prognosis, with a 27% increased risk of cardiovascular death compared to individuals with recorded HFrEF (Appendix Tables 5 and 7). Accordingly, specialist HF review and innovative approaches to enable initiation of pharmacological treatment amongst all patients diagnosed with HF during an inpatient admission may narrow the disparities we observed.^24^

It may also be that the incomplete recording of HF phenotype represents more than a disease-specific care gap, but a general marker of poor health or insufficient interaction and care with community services. It is possible that these individuals were not clinically suitable for more intensive investigation and therapy because of adverse prognostic markers from other diseases. We noted that adjusted for RAAS blockade and beta blocker prescription made little difference to the worse outcomes experienced by the unspecified HF phenotype cohort. This may suggest that unspecified HF is a ‘data phenotype’ in its own right and conveys a specific prognosis, and that these individuals have a range of non-HF mechanisms that contribute to poor outcomes.

Our findings have important clinical implications. First, the EHR phenotype of unspecified HF presents an actionable target for quality improvement initiatives. Primary care EHRs in the UK cover 98% of the population,^9^ thus the nationwide cohort of unspecified HF could be identified in routine practice and efforts made to optimise the characterisation of phenotype and medical therapy. A search of primary care EHRs at GP practice level could identify individuals with HF without a phenotype specification, who could then be prioritised for echocardiography to assess LVEF, which would consequently establish eligibility for pharmacological treatments as recommended by NICE and meet current Quality Outcomes Framework (QOF) indicators for HF.^25^ Second, incentivising the precise recording of HF phenotype in the community may facilitate a greater recognition of follow-through investigations and treatments that are required to improve the quality of life and prognosis. At present, QOF indicators only require the establishment of a HF register in primary care but HFpEF constitutes half of HF cases and has divergent management recommendations,^1^^,^^25^ so it may be more appropriate to establish separate HFrEF and HFpEF registers. Indeed, the 2022 ESC Quality Indicators for HF recommend that patients with HF are classified as HFrEF, HFmrEF or HFpEF.^26^ Third, incongruence between EHR coding vocabulary and guidelines may impact on delivery of care. ICD-10 codes for HF allow the description of HF by aetiology (hypertensive heart disease with congestive HF, ischaemic cardiomyopathy), anatomy (left ventricular failure), and presentation (hypertensive heart and renal disease with congestive HF). None of these descriptions fit with the current left ventricular ejection fraction (LVEF)-centric phenotypes ubiquitous across HF guidelines.^1^^,^^2^ Though Read codes do allow description of LVEF (left ventricular systolic dysfunction, HFpEF)—it is likely that coding in community EHRs after a hospital admission will generally be handled by non-healthcare professional clinical coders, who are unlikely to seek and record further phenotypic information. Accordingly important information, requisite for subsequent disease monitoring and management, can be lost in the transition from secondary care and primary care. Involvement of HF specialist community teams after hospital discharge improves care,^1^ and accessibility to this service may ameliorate the disparities in care we observed after hospital discharge. Future guidelines may also consider synergising definitions with the diagnostic vocabulary available in clinical practice, which may better enable implementation of recommendations.

We acknowledge the study limitations. First, the main reason for primary care to code medical information is for clinical care and administrative purposes not research, and this could lead to misclassification. However, previous studies have shown that a coded HF diagnosis in CPRD has a positive predictive value of 82%.^27^ Second, LVEF values were not available. Without objective ascertainment of left ventricular function, the relative ratio of HFpEF and HFrEF amongst the unclassified HF cohort is uncertain and so the true extent of guideline non-adherence cannot be quantified. Furthermore interobserver variability in echocardiographic assessment of LVEF in the real-world can lead to misclassification of HF phenotype. Third, secondary care records did not provide access to diagnostic investigations (such as ECGs and echocardiograms) so some investigations may have been conducted during the index admission but would not have been counted in this study. Nonetheless, we found shortfalls in provision of investigations for patients with unspecified HF across patients diagnosed in inpatient and outpatient settings. Fourth, there may be variation in the validity of HF diagnoses in secondary care that were the primary and non-primary reason for admission, and we do not have the ability to further adjudicate these cases. Fifth, we were not able to differentiate between HF cases that had been diagnosed in the secondary care outpatient setting to the primary care setting and, given the different levels of expertise and resources for investigations between these settings, care and outcomes may have differed. Sixth, reliable information on patients’ symptom burden was not available and limited our ability to investigate precise indications for certain therapies such as MRAs. Seventh, residual measured and unmeasured confounding may have influenced our findings. For example, we noted that adjustment for RAAS and beta blocker prescription made little difference to the worse outcomes experienced by the unspecified HF phenotype cohort, which may suggest that these individuals have a range of non-HF mechanisms that contribute to poor outcomes.

In conclusion, nine in ten community-dwelling patients with newly diagnosed HF do not have their phenotype recorded in community records. Compared to individuals with a specified HF phenotype, they receive different management and have a worse prognosis. Mitigating the prognostic and healthcare resource burden of HF requires accurate and consistent coding to improve care.

## Contributors

YMN, KN, RN, JW and CPG conceived the idea of the study. YMN undertook data extraction and statistical analysis. KN and RN verified the underlying data. YMN, KN, RN, JW and CPG interpreted the findings. YMN, KN and RN drafted the manuscript. AB, GCF, MCP, KR, JW and CPG critically reviewed the manuscript. YMN, KN, RN, JW and CPG had full access to all data in the study. All authors accept responsibility to submit for publication.

## Data sharing statement

CPRD data governance does not allow us to distribute or make available patient data directly to other parties. Data used in this study can be accessed through CPRD (https://cprd.com/) subject to protocol approval. The diagnostic code lists used in this study are available on reasonable request of the corresponding author.

## Declaration of interests

YMN reports a study grant from Bayer, outside the submitted work. CPG reports personal fees from AstraZeneca, Amgen, Bayer, Boehrinher-Ingelheim, Daiichi Sankyo, Vifor, Pharma, Menarini, Wondr Medical, Raisio Group and Oxford University Press. He has received educational and research grants from BMS, Abbott inc., the British Heart Foundation, National Institute of Health Research, Horizon 2020, and from the European Society of Cardiology, outside the submitted work. MCP reports grants from Boehringer Ingelheim, Roche, SQ Innovations, Astra Zeneca, Novartis, Novo Nordisk, Medtronic, Boston Scientific, and Pharmacosomos. He reports consulting fees and payments from Boehringer Ingelheim, AstraZeneca, Novartis, Novo Nordisk, Pharmacosomos, Abbvie, Bayer, Takeda, Corvia, Cardorentis, Seimens, and Vifor. He has participated on Data Safety Monitoring Boards and Advisory Boards for Teikoku and AstraZeneca and is Director of Global Clinical Trials Partners. AB reports a grant from AstraZeneca. GCF reports consulting fees from Abbott, Amgen, AstraZeneca, Bayer, Eli Lilly, Janssen, Medtronic, Merck, Novartis, Pfizer and Cytokinetics. All other authors declare no competing interests.
