# Supplementary material for: Prognosis, characteristics, and provision of care for patients with the unspecified heart failure electronic health record phenotype: a population-based linked cohort study of 95262 individuals
Source: eClinicalMedicine. 2023 Aug 25;63:102164. doi: 10.1016/j.eclinm.2023.102164 (PMC10474358; doi:10.1016/j.eclinm.2023.102164)
Supplement: Supplementary File [file mmc1.pdf]

Supplementary file

Title: Prognosis, characteristics and provision of care for patients with unspecified heart failure electronic health record phenotype: population-based linked cohort study of 95 262 individuals

Table of Contents

Supplementary methods *3*
Supplementary methods 3
Supplementary Table 1. Heart failure coding 4
Supplementary Table 2. Clinical codes used to identify patients with heart failure subtypes.. 6
Supplementary Table 3. Heart failure medications, as per European Society of Cardiology guideline during the study period 7
Supplementary results *8*
Supplementary Table 4. Baseline characteristics by heart failure phenotypes and diagnostic settings 8
Supplementary Table 5. Investigations and treatments by heart failure phenotypes and diagnostic settings 11
Supplementary Table 6. Association between heart failure phenotypes and outcomes by diagnostic settings 16
Supplementary Table 7. Sensitivity analysis for association between heart failure phenotypes and outcomes 18
Supplementary Figure 1. Study flow chart 19
Supplementary Figure 2. Definitions of essential care indicators 20
Supplementary Figure 3. Histogram of coding gap between heart failure diagnosis and phenotype 21
Supplementary references *22*

Supplementary methods
Supplementary methods

Patient characteristics
Frailty was ascertained on the date of heart failure (HF) diagnosis using the electronic frailty index (eFI), which includes 36 equally weighted deficit variables, based on Read codes.[1,2] All patients had HF, which is one of the deficits. The eFI is the number of deficits present as a proportion of the total possible and then categorized into fit (0–0·12), mild (>0·12–0·24), moderate (>0·24–0·36), or severe (>0·36) frailty. With the exception of polypharmacy (≥5 prescriptions in preceding 12-months using the first six characters of British National Formulary [BNF] code), deficits were identified if they were recorded at any time point in a patient's electric health record preceding their inclusion.[1]

Statistical analysis
For Poisson regression models, we checked the linearity of continuous predictors with logit of the response variable visually. We also checked Schoenfeld residuals plots and the assumption of proportional hazards was not violated.

Supplementary Table 1. Heart failure coding

	Code	Description
Read	G580000	Acute congestive heart failure
Read	G582.00	Acute heart failure
Read	G581000	Acute left ventricular failure
Read	8H2S.00	Admit heart failure emergency
Read	G581.11	Asthma - cardiac
Read	G211100	Benign hypertensive heart disease with CCF
Read	G580.14	Biventricular failure
Read	G58..11	Cardiac failure
Read	G58z.12	Cardiac failure NOS
Read	8B29.00	Cardiac failure therapy
Read	G580100	Chronic congestive heart failure
Read	G41z.11	Chronic cor pulmonale
Read	G580300	Compensated cardiac failure
Read	G580.11	Congestive cardiac failure
Read	G580.00	Congestive heart failure
Read	G580400	Congestive heart failure due to valvular disease
Read	662T.00	Congestive heart failure monitoring
Read	G580200	Decompensated cardiac failure
Read	585g.00	Echocardiogram shows left ventricular diastolic dysfunction
Read	585f.00	Echocardiogram shows left ventricular systolic dysfunction
Read	679W100	Education about deteriorating heart failure
Read	14A6.00	H/O: heart failure
Read	14AM.00	H/O: Heart failure in last year
Read	8CMK.00	Has heart failure management plan
Read	G58..00	Heart failure
Read	662p.00	Heart failure 6 month review
Read	662W.00	Heart failure annual review
Read	SP11111	Heart failure as a complication of care
Read	8CL3.00	Heart failure care plan discussed with patient
Read	8CMW800	Heart failure clinical pathway
Read	1O1..00	Heart failure confirmed
Read	679X.00	Heart failure education
Read	8HBE.00	Heart failure follow-up
Read	G58z.00	Heart failure NOS
Read	661M500	Heart failure self-management plan agreed
Read	G583.00	Heart failure with normal ejection fraction
Read	G583.12	Heart failure with preserved ejection fraction
Read	G583.11	HFNEF - heart failure with normal ejection fraction
Read	G234.00	Hyperten heart&renal dis+both(congestv)heart and renal fail
Read	G21z100	Hypertensive heart disease NOS with CCF
Read	G232.00	Hypertensive heart&renal dis wth (congestive) heart failure
Read	33BA.00	Impaired left ventricular function
Read	G581.13	Impaired left ventricular function
Read	G5yyD00	Left ventricular cardiac dysfunction
Read	G5yyA00	Left ventricular diastolic dysfunction
Read	G581.00	Left ventricular failure
Read	G5yy900	Left ventricular systolic dysfunction
Read	G210100	Malignant hypertensive heart disease with CCF
Read	662f.00	New York Heart Association classification - class I
Read	662g.00	New York Heart Association classification - class II
Read	662h.00	New York Heart Association classification - class III
Read	662i.00	New York Heart Association classification - class IV
Read	G5y4z00	Post cardiac operation heart failure NOS
Read	8CeC.00	Preferred place of care for next exacerbation heart failure
Read	G581.12	Pulmonary oedema - acute
Read	8HHz.00	Referral to heart failure exercise programme
Read	9N6T.00	Referred by heart failure nurse specialist

Read	G1yz100	Rheumatic left ventricular failure
Read	G580.12	Right heart failure
Read	G580.13	Right ventricular failure
Read	G584.00	Right ventricular failure
Read	9N2p.00	Seen by community heart failure nurse
Read	9N0k.00	Seen in heart failure clinic
Read	G58z.11	Weak heart
ICD-10	I50.0	Congestive heart failure
ICD-10	I50.1	Left ventricular failure
ICD-10	I50.9	Heart failure, unspecified
ICD-10	I11.0	Hypertensive heart disease with congestive heart failure
ICD-10	I25.5	Ischaemic cardiomyopathy
ICD-10	I13.2	Hypertensive heart and renal disease with both congestive heart failure and renal failure
ICD-10	I13.0	Hypertensive heart and renal disease with congestive heart failure

ICD=international classification of diseases.

Supplementary Table 2. Clinical codes used to identify patients with heart failure subtypes

	Code	Description
**HFpEF**		
Read	5853000	Echocardiogram normal
Read	585R.00	Echocardiogram normal
Read	585g.00	Echocardiogram shows left ventricular diastolic dysfunction
Read	585k.00	Echocardiogram shows normal left ventricular function
Read	G583.00	Heart failure with normal ejection fraction
Read	G583.12	Heart failure with preserved ejection fraction
Read	G583.11	HFNEF - heart failure with normal ejection fraction
Read	G5yyA00	Left ventricular diastolic dysfunction
**HFrEF**		
Read	585f.00	Echocardiogram shows left ventricular systolic dysfunction
Read	G581.13	Impaired left ventricular function
Read	33BA.00	Impaired left ventricular function
Read	G343.00	Ischaemic cardiomyopathy
Read	G5yyD00	Left ventricular cardiac dysfunction
Read	G5yy900	Left ventricular systolic dysfunction
Read	G554400	Primary dilated cardiomyopathy
Read	G55y.11	Secondary dilated cardiomyopathy
ICD-10	I25.5	Ischaemic cardiomyopathy
ICD-10	I42.0	Dilated cardiomyopathy

HFpEF=heart failure with preserved ejection fraction; HFrEF=heart failure with reduced ejection fraction; ICD=international classification of diseases.

Supplementary Table 3. Heart failure medications, as per European Society of Cardiology guideline during the study period

Drug name	1997[3]	2001[4]	2005[5]	2008[6]	2012[7]	2016[8]	2021[9]
**ACEI**							
Banazepril							
Captopril							
Cilazapril							
Enalapril							
Fosinopril							
Lisinopril							
Perindopril							
Quinapril							
Ramipril							
Trandolapril							
**ARB**							
Candesartan							
Irbesartan							
Losartan							
Telmisartan							
Valsartan							
**Beta blocker**							
Bisoprolol							
Carvedilol							
Metoprolol succinate							
Metoprolol tartrate							
Nebivolol							
**MRA**							
Eplerenone	-	-					
Spironolactone							
**ARNI**							
Sacubitril/valsartan							
**SGLT2 inhibitor**							
Dapagliflozin							
Empagliflozin							

Shade means guideline-recommended medications.
ACEI=angiotensin-converting-enzyme inhibitor; ARB=angiotensin receptor blocker; MRA=mineralocorticoid receptor antagonist; SGLT2 = sodium-glucose co-transporter 2.

Supplementary results

Supplementary Table 4. Baseline characteristics by heart failure phenotypes and diagnostic settings

Diagnostic setting	Outpatient (primary care)			Inpatient (secondary care)		
HF phenotype	HFrEF n=8263	HFpEF n=1009	Unspecified HF n=30129	HFrEF n=2530	HFpEF n=262	Unspecified HF n=53069
Age (years)	70.2 (12.8)	74.2 (10.9)	77.3 (10.9)	69.0 (14.4)	75.1 (12.3)	77.8 (12.1)
Sex						
Men	5432 (65.7%)	445 (44.1%)	15113 (50.2%)	1655 (65.4%)	135 (51.5%)	25635 (48.3%)
Women	2831 (34.3%)	564 (55.9%)	15016 (49.8%)	875 (34.6%)	127 (48.5%)	27434 (51.7%)
Ethnicity						
White	7617 (92.2%)	909 (90.1%)	25575 (84.9%)	2308 (91.2%)	237 (90.5%)	47510 (89.5%)
Others	342 (4.1%)	69 (6.8%)	903 (3.0%)	123 (4.9%)	18 (6.9%)	1904 (3.6%)
Missing	304 (3.7%)	31 (3.1%)	3651 (12.1%)	99 (3.9%)	7 (2.7%)	3655 (6.9%)
Socioeconomic status quintile						
1 (least deprived)	1516 (18.3%)	183 (18.1%)	5321 (17.7%)	477 (18.9%)	25 (9.5%)	8999 (17.0%)
2	1629 (19.7%)	194 (19.2%)	6169 (20.5%)	448 (17.7%)	42 (16.0%)	10171 (19.2%)
3	1893 (22.9%)	234 (23.2%)	6665 (22.1%)	574 (22.7%)	50 (19.1%)	11593 (21.8%)
4	1697 (20.5%)	200 (19.8%)	6518 (21.6%)	533 (21.1%)	74 (28.2%)	11634 (21.9%)
5 (most deprived)	1525 (18.5%)	198 (19.6%)	5433 (18.0%)	495 (19.6%)	71 (27.1%)	10646 (20.1%)
Missing	3 (<1%)	0 (0.0%)	23 (0.1%)	3 (0.1%)	0 (0.0%)	26 (<1%)
Smoking						
Ever	5252 (63.6%)	583 (57.8%)	15239 (50.6%)	1582 (62.5%)	165 (63.0%)	28825 (54.3%)
No	1494 (18.1%)	227 (22.5%)	3959 (13.1%)	407 (16.1%)	54 (20.6%)	7176 (13.5%)
Missing	1517 (18.4%)	199 (19.7%)	10931 (36.3%)	541 (21.4%)	43 (16.4%)	17068 (32.2%)
BMI (kg/m^2)	29.0 (6.1)	29.7 (6.3)	28.6 (6.5)	28.8 (6.5)	30.2 (6.9)	28.2 (6.9)
BMI category						
Underweight	71 (0.9%)	8 (0.8%)	319 (1.1%)	19 (0.8%)	4 (1.5%)	870 (1.6%)
Normal	1105 (13.4%)	126 (12.5%)	3126 (10.4%)	321 (12.7%)	35 (13.4%)	6049 (11.4%)
Overweight	1631 (19.7%)	182 (18.0%)	3916 (13.0%)	423 (16.7%)	44 (16.8%)	6324 (11.9%)

Obesity class I	1050 (12.7%)	147 (14.6%)	2394 (7.9%)	264 (10.4%)	34 (13.0%)	3776 (7.1%)
Obesity class II/III	629 (7.6%)	102 (10.1%)	1638 (5.4%)	169 (6.7%)	34 (13.0%)	2859 (5.4%)
Missing	3777 (45.7%)	444 (44.0%)	18736 (62.2%)	1334 (52.7%)	111 (42.4%)	33191 (62.5%)
Cardiovascular comorbidities						
Atrial fibrillation	2674 (32.4%)	257 (25.5%)	9064 (30.1%)	992 (39.2%)	107 (40.8%)	21227 (40.0%)
Hypertension	5219 (63.2%)	752 (74.5%)	17896 (59.4%)	1606 (63.5%)	195 (74.4%)	36071 (68.0%)
Ischaemic heart disease	4211 (51.0%)	398 (39.4%)	12259 (40.7%)	1404 (55.5%)	133 (50.8%)	27895 (52.6%)
Stroke	1022 (12.4%)	139 (13.8%)	4634 (15.4%)	299 (11.8%)	42 (16.0%)	10011 (18.9%)
Valvular heart disease	1439 (17.4%)	185 (18.3%)	3342 (11.1%)	668 (26.4%)	55 (21.0%)	9927 (18.7%)
Non-cardiovascular comorbidities						
Anaemia	1513 (18.3%)	235 (23.3%)	5754 (19.1%)	555 (21.9%)	81 (30.9%)	15673 (29.5%)
Cancer	1146 (13.9%)	151 (15.0%)	4154 (13.8%)	385 (15.2%)	50 (19.1%)	10166 (19.2%)
Chronic kidney disease	1019 (12.3%)	166 (16.5%)	2832 (9.4%)	420 (16.6%)	58 (22.1%)	10682 (20.1%)
COPD	1175 (14.2%)	158 (15.7%)	5051 (16.8%)	442 (17.5%)	57 (21.8%)	12024 (22.7%)
Dementia	135 (1.6%)	20 (2.0%)	1070 (3.6%)	54 (2.1%)	4 (1.5%)	3995 (7.5%)
Depression	1712 (20.7%)	234 (23.2%)	5113 (17.0%)	529 (20.9%)	55 (21.0%)	10547 (19.9%)
Diabetes	1831 (22.2%)	222 (22.0%)	5850 (19.4%)	605 (23.9%)	87 (33.2%)	13210 (24.9%)
Dyslipidaemia	2841 (34.4%)	348 (34.5%)	5913 (19.6%)	895 (35.4%)	115 (43.9%)	14715 (27.7%)
Gout	820 (9.9%)	98 (9.7%)	2588 (8.6%)	251 (9.9%)	23 (8.8%)	4986 (9.4%)
Sleep apnoea syndrome	137 (1.7%)	24 (2.4%)	230 (0.8%)	57 (2.3%)	5 (1.9%)	762 (1.4%)
Thyroid disease	769 (9.3%)	143 (14.2%)	3085 (10.2%)	226 (8.9%)	30 (11.5%)	6520 (12.3%)
Three or more comorbidities	5149 (62.3%)	651 (64.5%)	16321 (54.2%)	1740 (68.8%)	199 (76.0%)	38620 (72.8%)
Frailty						
Fit	2132 (25.8%)	150 (14.9%)	6822 (22.6%)	803 (31.7%)	43 (16.4%)	11823 (22.3%)
Mild frailty	3723 (45.1%)	469 (46.5%)	14109 (46.8%)	1055 (41.7%)	108 (41.2%)	20851 (39.3%)
Moderate frailty	1852 (22.4%)	298 (29.5%)	7169 (23.8%)	519 (20.5%)	76 (29.0%)	14747 (27.8%)
Severe frailty	556 (6.7%)	92 (9.1%)	2029 (6.7%)	153 (6.0%)	35 (13.4%)	5648 (10.6%)

Mean (standard deviation) or number (%).

BMI=body mass index; HF=heart failure; COPD=chronic obstructive pulmonary disease HFpEF=heart failure with preserved ejection fraction; HFrEF=heart failure with reduced ejection fraction.

Supplementary Table 5. Investigations and treatments by heart failure phenotypes and diagnostic settings

	HFrEF	HFpEF	Unspecified HF	Risk ratio (95% CI)	
				HFpEF	Unspecified HF
**Outpatient (primary care)**					
n	8263	1009	30129		
Diagnostic investigation					
Echocardiogram	6082 (73.6%)	717 (71.1%)	8142 (27.0%)	1.00 (0.95 – 1.04)	0.61 (0.60 – 1.04)
ECG	3015 (36.5%)	373 (37.0%)	6412 (21.3%)	1.04 (0.96 – 1.14)	0.87 (0.83 – 0.90)
NP test	814 (9.9%)	139 (13.8%)	2369 (7.9%)	1.24 (1.44 – 1.68)	1.56 (1.44 – 1.68)
Chest radiography	1813 (21.9%)	228 (22.6%)	6458 (21.4%)	1.04 (0.92 – 1.17)	1.23 (1.17 – 1.29)
Other blood tests					
Full blood count	1068 (12.9%)	126 (12.5%)	4122 (13.7%)	0.95 (0.81 – 1.12)	1.11 (1.04 – 1.19)
Urea and/or electrolytes	2551 (30.9%)	331 (32.8%)	8147 (27.0%)	1.03 (0.94 – 1.13)	1.12 (1.07 – 1.16)
Thyroid function	2116 (25.6%)	224 (22.2%)	5671 (18.8%)	0.82 (0.72 – 0.93)	1.01 (0.96 – 1.06)
Fasting glucose/HbA1c,	2297 (27.8%)	267 (26.5%)	6159 (20.4%)	0.96 (0.86 – 1.07)	1.07 (1.03 – 1.12)
Lipids	2202 (26.6%)	249 (24.7%)	5062 (16.8%)	0.99 (0.89 – 1.12)	0.99 (0.94 – 1.04)
Iron status	596 (7.2%)	90 (8.9%)	1501 (5.0%)	1.12 (0.91 – 1.38)	1.19 (1.09 – 1.31)
All blood tests above	28 (0.3%)	4 (0.4%)	75 (0.2%)	1.15 (0.40 – 3.29)	1.20 (0.75 – 1.92)
At least 1 diagnostic investigation	6888 (83.4%)	838 (83.1%)	14640 (48.6%)	1.02 (0.99 – 1.05)	0.84 (0.82 – 0.85)
Specialist assessment	1109 (13.4%)	147 (14.6%)	1981 (6.6%)	1.12 (0.95 – 1.31)	0.88 (0.82 – 0.95)
Treatment initiation					
Within 3 months					
ACEI	5807 (70.3%)	500 (49.6%)	15402 (51.1%)	0.75 (0.70 – 0.80)	0.82 (0.80 – 0.84)
ARB	1184 (14.3%)	189 (18.7%)	3033 (10.1%)	1.25 (1.09 – 1.44)	0.99 (0.93 – 1.06)
ARNI	13 (0.2%)	0 (0.0%)	4 (<1%)	-	-
Beta blocker	4403 (53.3%)	311 (30.8%)	6130 (20.3%)	0.61 (0.56 – 0.67)	0.75 (0.73 – 0.78)
Diuretics	4476 (54.2%)	607 (60.2%)	22996 (76.3%)	1.06 (1.01 – 1.12)	1.24 (1.21 – 1.27)
MRA	1423 (17.2%)	108 (10.7%)	3373 (11.2%)	0.66 (0.55 – 0.79)	1.00 (0.94 – 1.06)

SGLT2i	21 (0.3%)	2 (0.2%)	24 (0.1%)	1.11 (0.24 – 5.08)	0.79 (0.43 – 1.46)
Within 6 months					
ACEI	6083 (73.6%)	532 (52.7%)	16355 (54.3%)	0.76 (0.71 – 0.80)	0.82 (0.80 – 0.83)
ARB	1425 (17.2%)	206 (20.4%)	3520 (11.7%)	1.14 (1.00 – 1.30)	0.93 (0.87 – 0.99)
ARNI	29 (0.4%)	0 (0.0%)	7 (<1%)	-	-
Beta blocker	4823 (58.4%)	346 (34.3%)	6764 (22.5%)	0.62 (0.57 – 0.68)	0.74 (0.72 – 0.76)
Diuretics	4757 (57.6%)	652 (64.6%)	24019 (79.7%)	1.08 (1.03 – 1.13)	1.22 (1.20 – 1.24)
MRA	1710 (20.7%)	135 (13.4%)	4018 (13.3%)	0.68 (0.58 – 0.80)	0.97 (0.92 – 1.02)
SGLT2i	32 (0.4%)	3 (0.3%)	31 (0.1%)	1.01 (0.29 – 3.53)	0.63 (0.37 – 1.06)
Within 12 months					
ACEI	6301 (76.3%)	559 (55.4%)	17249 (57.3%)	0.77 (0.72 – 0.81)	0.82 (0.81 – 0.84)
ARB	1674 (20.3%)	228 (22.6%)	4043 (13.4%)	1.09 (0.97 – 1.23)	0.89 (0.84 – 0.95)
ARNI	50 (0.6%)	0 (0.0%)	19 (0.1%)	-	-
Beta blocker	5142 (62.2%)	367 (36.4%)	7417 (24.6%)	0.62 (0.57 – 0.67)	0.74 (0.72 – 0.76)
Diuretics	5039 (61.0%)	688 (68.2%)	24749 (82.1%)	1.08 (1.03 – 1.12)	1.19 (1.17 – 1.21)
MRA	2041 (24.7%)	151 (15.0%)	4749 (15.8%)	0.64 (0.55 – 0.75)	0.93 (0.88 – 0.98)
SGLT2i	41 (0.5%)	3 (0.3%)	38 (0.1%)	0.77 (0.23 – 2.62)	0.62 (0.39 – 1.00)
**Inpatient (secondary care)**					
n	2530	262	53069		
Diagnostic investigation					
Echocardiogram	1454 (57.5%)	175 (66.8%)	5690 (10.7%)	1.22 (1.11 – 1.35)	0.24 (0.23 – 0.25)
ECG	752 (29.7%)	70 (26.7%)	6771 (12.8%)	0.96 (0.78 – 1.19)	0.55 (0.51 – 0.58)
NP test	142 (5.6%)	16 (6.1%)	1328 (2.5%)	1.20 (0.73 – 1.96)	0.63 (0.53 – 0.75)
Chest radiography	530 (20.9%)	49 (18.7%)	6621 (12.5%)	0.92 (0.71 – 1.20)	0.70 (0.64 – 0.76)
Other blood tests					
Full blood count	291 (11.5%)	26 (9.9%)	6529 (12.3%)	0.67 (0.46 – 0.96)	0.94 (0.85 – 1.05)
Urea and/or electrolytes	669 (26.4%)	81 (30.9%)	14118 (26.6%)	1.11 (0.92 – 1.34)	1.06 (0.99 – 1.13)
Thyroid function	496 (19.6%)	51 (19.5%)	8601 (16.2%)	0.95 (0.74 – 1.23)	0.89 (0.82 – 0.97)
Fasting glucose/HbA1c,	609 (24.1%)	68 (26.0%)	10299 (19.4%)	1.07 (0.86 – 1.33)	0.93 (0.87 – 1.00)
Lipids	507 (20.0%)	62 (23.7%)	7842 (14.8%)	1.23 (0.98 – 1.55)	0.92 (0.85 – 1.00)

Iron status	200 (7.9%)	23 (8.8%)	3409 (6.4%)	1.09 (0.73 – 1.63)	0.95 (0.83 – 1.09)
All blood tests above	7 (0.3%)	1 (0.4%)	191 (0.4%)	1.13 (0.14 – 9.21)	1.41 (0.66 – 3.02)
At least 1 diagnostic investigation	1780 (70.4%)	200 (76.3%)	14654 (27.6%)	1.14 (1.06 – 1.23)	0.48 (0.47 – 0.50)
Specialist assessment	204 (8.1%)	13 (5.0%)	1500 (2.8%)	0.75 (0.43 – 1.30)	0.52 (0.45 – 0.60)
Treatment initiation					
Within 3 months					
ACEI	1705 (67.4%)	125 (47.7%)	17640 (33.2%)	0.78 (0.69 – 0.89)	0.57 (0.55 – 0.59)
ARB	322 (12.7%)	43 (16.4%)	3913 (7.4%)	1.41 (1.05 – 1.88)	0.73 (0.65 – 0.81)
ARNI	10 (0.4%)	0 (0.0%)	10 (<1%)	-	
Beta blocker	1571 (62.1%)	103 (39.3%)	11145 (21.0%)	0.77 (0.67 – 0.89)	0.51 (0.49 – 0.53)
Diuretics	1808 (71.5%)	177 (67.6%)	26547 (50.0%)	0.91 (0.83 – 0.99)	0.66 (0.64 – 0.67)
MRA	860 (34.0%)	39 (14.9%)	4403 (8.3%)	0.49 (0.36 – 0.65)	0.31 (0.29 – 0.33)
SGLT2i	9 (0.4%)	0 (0.0%)	35 (0.1%)	-	
Within 6 months					
ACEI	1831 (72.4%)	135 (51.5%)	18946 (35.7%)	0.78 (0.70 – 0.88)	0.56 (0.55 – 0.59)
ARB	390 (15.4%)	50 (19.1%)	4450 (8.4%)	1.36 (1.04 – 1.77)	0.68 (0.61 – 0.75)
ARNI	15 (0.6%)	0 (0.0%)	13 (<1%)	-	
Beta blocker	1682 (66.5%)	112 (42.7%)	11987 (22.6%)	0.78 (0.68 – 0.89)	0.51 (0.49 – 0.52)
Diuretics	1899 (75.1%)	189 (72.1%)	28516 (53.7%)	0.92 (0.86 – 1.00)	0.67 (0.65 – 0.69)
MRA	983 (38.9%)	46 (17.6%)	5198 (9.8%)	0.50 (0.38 – 0.65)	0.31 (0.29 – 0.33)
SGLT2i	13 (0.5%)	0 (0.0%)	45 (0.1%)	-	
Within 12 months					
ACEI	1876 (74.2%)	140 (53.4%)	19876 (37.5%)	0.79 (0.71 – 0.89)	0.57 (0.56 – 0.59)
ARB	464 (18.3%)	54 (20.6%)	4975 (9.4%)	1.24 (0.97 – 1.60)	0.63 (0.58 – 0.69)
ARNI	30 (1.2%)	0 (0.0%)	25 (<1%)	-	
Beta blocker	1766 (69.8%)	116 (44.3%)	12712 (24.0%)	0.77 (0.67 – 0.87)	0.51 (0.49 – 0.52)
Diuretics	1943 (76.8%)	193 (73.7%)	29508 (55.6%)	0.93 (0.86 – 1.00)	0.68 (0.66 – 0.69)
MRA	1064 (42.1%)	52 (19.8%)	5930 (11.2%)	0.52 (0.40 – 0.66)	0.33 (0.31 – 0.34)
SGLT2i	18 (0.7%)	0 (0.0%)	55 (0.1%)	-	

**Inpatient (secondary care), HF primary cause**					
n	973	96	12831		
Diagnostic investigation					
Echocardiogram	572 (58.8%)	73 (76.0%)	2004 (15.6%)	1.36 (1.19 – 1.57)	0.37 (0.35 – 0.40)
ECG	296 (30.4%)	26 (27.1%)	1827 (14.2%)	0.97 (0.69 – 1.35)	0.64 (0.58 – 0.72)
NP test	72 (7.4%)	7 (7.3%)	450 (3.5%)	1.15 (0.58 – 2.30)	0.85 (0.67 – 1.09)
Chest radiography	251 (25.8%)	21 (21.9%)	1832 (14.3%)	0.91 (0.61 – 1.35)	0.74 (0.65 – 0.83)
Other blood tests					
Full blood count	126 (12.9%)	11 (11.5%)	1757 (13.7%)	0.71 (0.41 – 1.22)	0.97 (0.82 – 1.14)
Urea and/or electrolytes	277 (28.5%)	32 (33.3%)	3618 (28.2%)	1.13 (0.84 – 1.53)	1.11 (1.00 – 1.24)
Thyroid function	218 (22.4%)	18 (18.8%)	2262 (17.6%)	0.81 (0.53 – 1.24)	0.91 (0.80 – 1.04)
Fasting glucose/HbA1c,	253 (26.0%)	24 (25.0%)	2564 (20.0%)	0.98 (0.68 – 1.41)	0.97 (0.86 – 1.09)
Lipids	202 (20.8%)	22 (22.9%)	1874 (14.6%)	1.17 (0.80 – 1.73)	0.95 (0.83 – 1.09)
Iron status	98 (10.1%)	8 (8.3%)	870 (6.8%)	0.82 (0.42 – 1.61)	0.92 (0.75 – 1.12)
All blood tests above	3 (0.3%)	0 (0.0%)	55 (0.4%)	-	-
At least 1 diagnostic investigation	711 (73.1%)	80 (83.3%)	4218 (32.9%)	1.22 (1.10 – 1.36)	0.61 (0.58 – 0.64)
Specialist assessment	92 (9.5%)	2 (2.1%)	441 (3.4%)	0.29 (0.07 – 1.17)	0.63 (0.50 – 0.80)
Treatment initiation					
Within 3 months					
ACEI	656 (67.4%)	43 (44.8%)	5062 (39.5%)	0.73 (0.58 – 0.91)	065 (0.61 – 0.68)
ARB	133 (13.7%)	23 (24.0%)	1045 (8.1%)	2.06 (1.39 – 3.05)	0.86 (0.72 – 1.02)
ARNI	2 (0.2%)	0 (0.0%)	3 (<1%)	-	-
Beta blocker	590 (60.6%)	36 (37.5%)	2754 (21.5%)	0.79 (0.62 – 1.00)	0.59 (0.55 – 0.63)
Diuretics	798 (82.0%)	76 (79.2%)	8036 (62.6%)	0.98 (0.88 – 1.09)	0.79 (0.77 – 0.82)
MRA	378 (38.8%)	15 (15.6%)	1668 (13.0%)	0.47 (0.29 – 0.76)	0.46 (0.42 – 0.51)
SGLT2i	3 (0.3%)	0 (0.0%)	7 (0.1%)	-	-
Within 6 months					
ACEI	708 (72.8%)	48 (50.0%)	5357 (41.8%)	0.75 (0.61 – 0.92)	0.62 (0.60 – 0.66)
ARB	156 (16.0%)	26 (27.1%)	1196 (9.3%)	1.98 (1.38 – 2.84)	0.82 (0.70 – 0.97)
ARNI	6 (0.6%)	0 (0.0%)	3 (<1%)	-	-

Beta blocker	637 (65.5%)	41 (42.7%)	2967 (23.1%)	0.83 (0.67 – 1.02)	0.58 (0.55 – 0.62)
Diuretics	822 (84.5%)	78 (81.3%)	8422 (65.6%)	0.98 (0.88 – 1.08)	0.80 (0.78 – 0.83)
MRA	422 (43.4%)	16 (16.7%)	1928 (15.0%)	0.45 (0.28 – 0.70)	0.47 (0.43 – 0.51)
SGLT2i	6 (0.6%)	0 (0.0%)	9 (0.1%)	-	-
Within 12 months					
ACEI	721 (74.1%)	50 (52.1%)	5560 (43.3%)	0.77 (0.63 – 0.93)	0.63 (0.60 – 0.66)
ARB	180 (18.5%)	27 (28.1%)	1346 (10.5%)	1.79 (1.26 – 2.54)	0.79 (0.68 – 0.92)
ARNI	14 (1.4%)	0 (0.0%)	10 (0.1%)	-	-
Beta blocker	674 (69.3%)	44 (45.8%)	3162 (24.6%)	0.84 (0.69 – 1.03)	0.58 (0.55 – 0.61)
Diuretics	837 (86.0%)	79 (82.3%)	8558 (66.7%)	0.98 (0.88 – 1.08)	0.80 (0.77 – 0.82)
MRA	447 (45.9%)	19 (19.8%)	2167 (16.9%)	0.50 (0.33 – 0.75)	0.49 (0.45 – 0.54)
SGLT2i	9 (0.9%)	0 (0.0%)	11 (0.1%)	-	-

RRs and 95% CIs comparing HFpEF and unspecified HF to HFrEF (reference), adjusting for year of diagnosis, age, sex and socioeconomic status. ACEI=angiotensin-converting-enzyme inhibitor; ARB=angiotensin receptor blocker; ARNI= Angiotensin Receptor-Neprilysin Inhibitor; CI=confidence interval; ECG=electrocardiogram; HF=heart failure; HFpEF=heart failure with preserved ejection fraction; HFrEF=heart failure with reduced ejection fraction; MRA=mineralocorticoid receptor antagonist; NP=natriuretic peptide; RR = risk ratio; SGLT2i=Sodium-glucose cotransporter-2 inhibitors.

Supplementary Table 6. Association between heart failure phenotypes and outcomes by diagnostic settings

	Outpatient (primary care)				Inpatient (secondary care)				Inpatient, HF primary cause			
	Number of events	Crude incidence per 100 person-years (95% CI)	Adjusted HR (95% CI)	P value	Number of events	Crude incidence per 100 person-years (95% CI)	Adjusted HR (95% CI)	P value	Number of events	Crude incidence per 100 person-years (95% CI)	Adjusted HR (95% CI)	P value
**Primary outcome**												
HFrEF	3144	8.4 (8.1 – 8.7)	Reference		1258	15.6 (13.2 – 18.5)	Reference		536	18.2 (16.7 – 19.8)	Reference	
HFpEF	343	7.9 (7.1 – 8.7)	0.83 (0.74 – 0.93)	<0.01	137	15.3 (14.4 – 16.1)	0.83 (0.70 – 0.99)	0.04	56	17.1 (13.2 – 22.2)	0.77 (0.58 – 1.01)	0.06
Unspecified HF	19305	17.8 (17.6 – 18.1)	1.61 (1.55 – 1.67)	<0.01	35311	29.7 (29.4 – 30.0)	1.31 (1.24 – 1.39)	<0.01	9338	34.8 (34.1 – 35.5)	1.34 (1.23 – 1.46)	<0.01
**Secondary outcome**												
HF hospitalisation												
HFrEF	1433	3.8 (3.6 – 4.0)	Reference		620	7.5 (6.9 – 8.1)	Reference		289	9.9 (8.7 – 11.0)	Reference	
HfpEF	99	2.3 (1.9 – 2.8)	0.54 (0.44 – 0.67)	<0.01	50	5.7 (4.3 – 7.5)	0.64 (0.48 – 0.86)	<0.01	23	7.0 (4.7 – 10.6)	0.61 (0.40 – 0.94)	0.03
Unspecified HF	5539	5.1 (5.0 – 5.3)	1.18 (1.11 – 1.26)	<0.01	6830	5.7 (5.6 – 5.9)	0.61 (0.57 – 0.67)	<0.01	2659	9.9 (9.5 – 10.3)	0.82 (0.72 – 0.93)	<0.01
All-cause mortality												

HFrEF	2412	5.8 (6.0 – 6.1)	Reference		958	9.6 (9.0 – 10.2)	Reference		407	10.8 (9.8 – 11.9)	Reference	
HFpEF	307	6.7 (6.0 – 7.5)	1.01 (0.89 – 1.13)	0.92	116	11.8 (9.8 – 14.2)	0.97 (0.80 – 1.18)	0.78	46	12.1 (9.1 – 16.2)	0.89 (0.66 – 1.21)	0.46
Unspecified HF	17812	14.9 (14.6 – 15.1)	1.87 (1.79 – 1.95)	<0.01	33337	25.5 (25.2 – 25.8)	1.72 (1.61 – 1.83)	<0.01	8592	27.3 (26.7 – 27.9)	1.71 (1.54 – 1.89)	<0.01
Cardiovascular death												
HFrEF	1218	2.9 (2.8 – 3.1)	Reference		510	5.1 (4.7 – 5.5)	Reference		223	5.9 (5.2 – 6.7)	Reference	
HfpEF	105	2.9 (2.4 – 3.4)	0.89 (0.75 – 1.07)	0.21	53	5.4 (4.1 – 7.1)	0.85 (0.64 – 1.12)	0.25	27	7.1 (4.9 – 18.4)	0.93 0.63 – 1.38)	0.71
Unspecified HF	7881	6.6 (6.4 – 6.7)	1.76 (1.65 – 1.87)	<0.01	13926	10.7 (10.5 – 10.8)	1.36 (1.24 – 1.48)	<0.01	4297	13.6 (13.2 – 14.1)	1.53 (1.33 – 1.75)	<0.01

Model was adjusted for age, sex, ethnicity, socioeconomic status, smoking, body mass index, atrial fibrillation, hypertension, ischaemic heart disease, stroke, valvular heart disease, anaemia, cancer, chronic kidney disease, chronic obstructive pulmonary disease, dementia, depression, diabetes, dyslipidaemia, gout, sleep apnoea syndrome and thyroid disease. CI=confidence interval; HF=heart failure; HR=hazard ratio; HFpEF=heart failure with preserved ejection fraction; HFrEF=heart failure with reduced ejection fraction.

Supplementary Table 7. Sensitivity analysis for association between heart failure phenotypes and outcomes

	Adjusted HR (95% CI)	P value
**Ethnicity and smoking***		
**Primary outcome**		
HFrEF	Reference	
HFpEF	0.84 (0.76 – 0.92)	<0.01
Unspecified HF	1.69 (1.64 – 1.75)	<0.01
**Secondary outcomes**		
HF hospitalisation		
HFrEF	Reference	
HFpEF	0.58 (0.49 – 0.68)	<0.01
Unspecified HF	0.98 (0.93 – 1.03)	0.38
All-cause mortality		
HFrEF	Reference	
HFpEF	0.98 (0.90 – 1.10)	0.96
Unspecified HF	2.06 (1.99 – 2.13)	<0.01
Cardiovascular death		
HFrEF	Reference	
HFpEF	0.87 (0.75 – 1.01)	0.08
Unspecified HF	1.77 (1.69 – 1.86)	<0.01
**Further adjustment for ACEI, ARB, ARNI and beta-blockers**		
**Primary outcome**		
HFrEF	Reference	
HFpEF	0.83 (0.75 – 0.91)	<0.01
Unspecified HF	1.68 (1.63 – 1.74)	<0.01
**Secondary outcomes**		
HF hospitalisation		
HFrEF	Reference	
HFpEF	0.57 (0.48 – 0.68)	<0.01
Unspecified HF	0.97 (0.93 – 1.02)	0.29
All-cause mortality		
HFrEF	Reference	
HFpEF	0.99 (0.89 – 1.09)	0.79
Unspecified HF	2.05 (1.98 – 2.12)	<0.01
Cardiovascular death		
HFrEF	Reference	
HFpEF	0.86 (0.74 – 1.00)	0.06
Unspecified HF	1.77 (1.68 – 1.86)	<0.01

*A sensitivity analysis was run for ethnicity and smoking where the missing values were included as a separate category. Model was adjusted for age, sex, ethnicity (white, others, missing), socioeconomic status, smoking (ever, no, missing), body mass index, atrial fibrillation, hypertension, ischaemic heart disease, stroke, valvular heart disease, anaemia, cancer, chronic kidney disease, chronic obstructive pulmonary disease, dementia, depression, diabetes, dyslipidaemia, gout, sleep apnoea syndrome and thyroid disease.

ACEI: angiotensin-converting-enzyme inhibitor; ARB: angiotensin receptor blocker; ARNI: angiotensin receptor-neprilysin inhibitor; CI=confidence interval; HF=heart failure; HR=hazard ratio; HFpEF=heart failure with preserved ejection fraction; HFrEF=heart failure with reduced ejection fraction

Supplementary Figure 1. Study flow chart

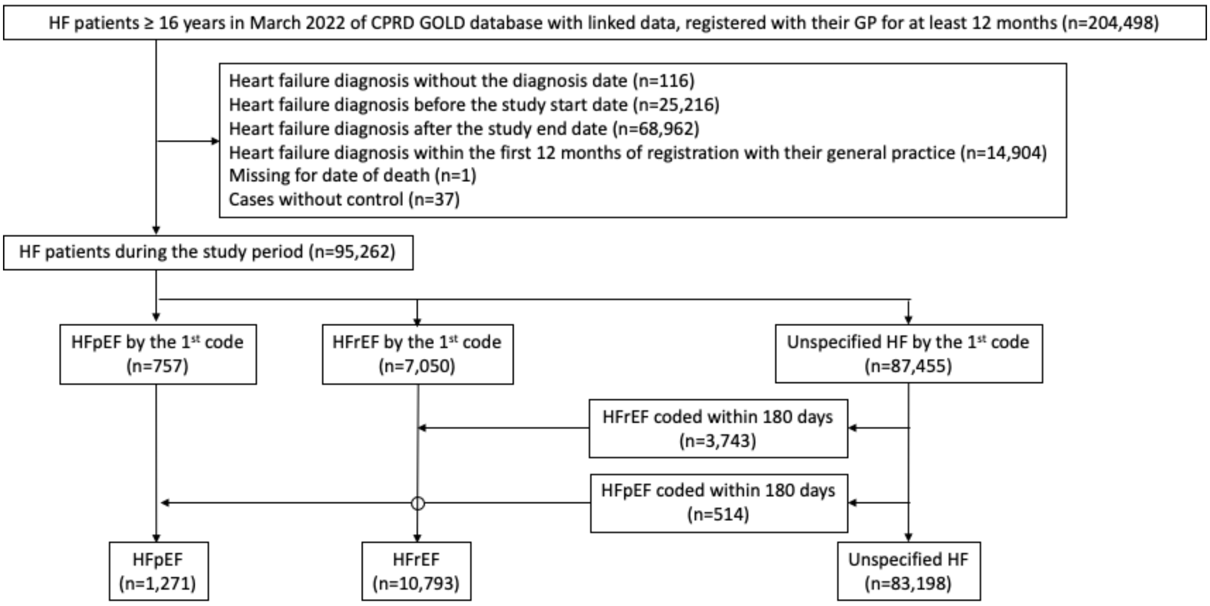

CPRD= Clinical Practice Research Datalink; GP=general practice; HF=heart failure; HFpEF=heart failure with preserved ejection fraction; HFrEF=heart failure with reduced ejection fraction.

Supplementary Figure 2. Definitions of essential care indicators

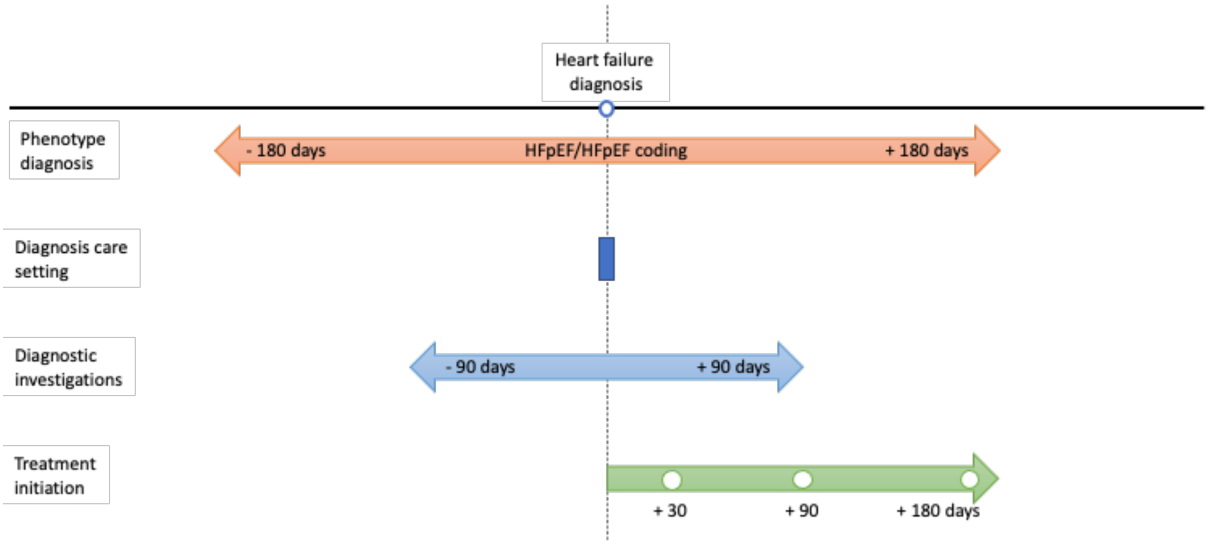

HF=heart failure; HFpEF=heart failure with preserved ejection fraction; HFrEF=heart failure with reduced ejection fraction.

Supplementary Figure 3. Histogram of coding gap between heart failure diagnosis and phenotype

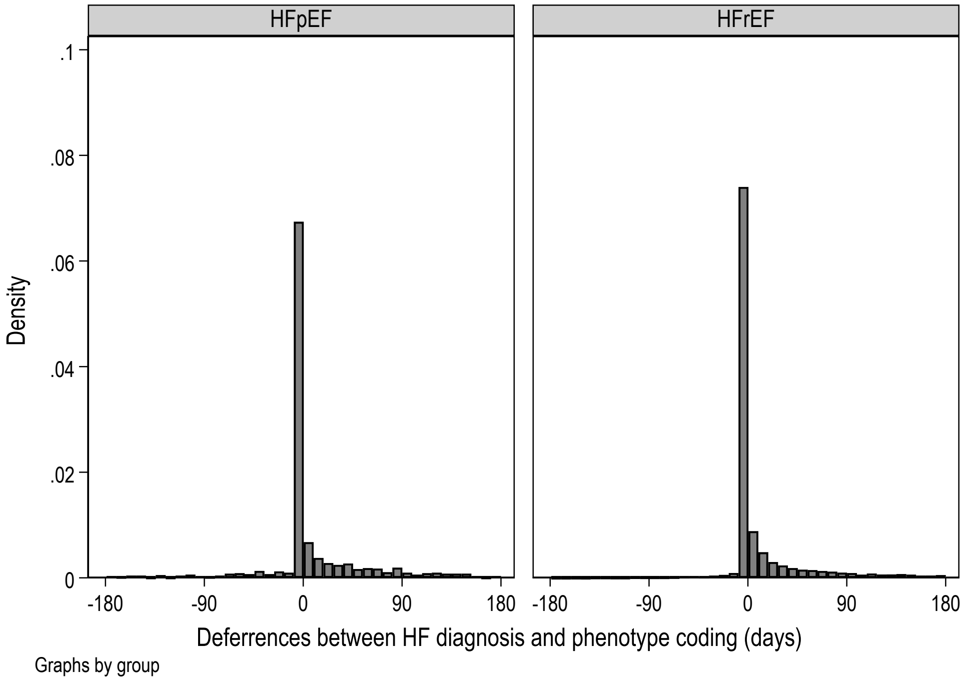

Median (interquartile range) was 0 (0 – 5) days in heart failure with preserved ejection fraction and 0 (0 – 8) days in heart failure with reduced ejection fraction.
HF=heart failure; HFpEF=heart failure with preserved ejection fraction; HFrEF=heart failure with reduced ejection fraction

Supplementary references

1. Clegg A, Bates C, Young J, et al. Development and validation of an electronic frailty index using routine primary care electronic health record data. *Age and Ageing* 2016; **45**(3): 353-60.
2. Wilkinson C, Wu J, Clegg A, et al. Impact of oral anticoagulation on the association between frailty and clinical outcomes in people with atrial fibrillation: nationwide primary care records on treatment analysis. *EP Europace* 2022; **24**(7): 1065-75.
3. The treatment of heart failure: The Task Force of the Working Group on Heart Failure of the European Society of Cardiology. *European Heart Journal* 1997; **18**(5): 736-53.
4. Remme WJ, Swedberg K. Guidelines for the diagnosis and treatment of chronic heart failure. *Eur Heart J* 2001; **22**(17): 1527-60.
5. Swedberg K, Cleland J, Dargie H, et al. Guidelines for the diagnosis and treatment of chronic heart failure: executive summary (update 2005): The Task Force for the Diagnosis and Treatment of Chronic Heart Failure of the European Society of Cardiology. *Eur Heart J* 2005; **26**(11): 1115-40.
6. Dickstein K, Cohen-Solal A, Filippatos G, et al. ESC guidelines for the diagnosis and treatment of acute and chronic heart failure 2008: the Task Force for the diagnosis and treatment of acute and chronic heart failure 2008 of the European Society of Cardiology. Developed in collaboration with the Heart Failure Association of the ESC (HFA) and endorsed by the European Society of Intensive Care Medicine (ESICM). *Eur J Heart Fail* 2008; **10**(10): 933-89.
7. McMurray JJ, Adamopoulos S, Anker SD, et al. ESC guidelines for the diagnosis and treatment of acute and chronic heart failure 2012: The Task Force for the Diagnosis and Treatment of Acute and Chronic Heart Failure 2012 of the European Society of Cardiology. Developed in collaboration with the Heart Failure Association (HFA) of the ESC. *Eur J Heart Fail* 2012; **14**(8): 803-69.
8. Ponikowski P, Voors AA, Anker SD, et al. 2016 ESC Guidelines for the diagnosis and treatment of acute and chronic heart failure: The Task Force for the diagnosis and treatment of acute and chronic heart failure of the European Society of Cardiology (ESC)Developed with the special contribution of the Heart Failure Association (HFA) of the ESC. *Eur Heart J* 2016; **37**(27): 2129-200.
9. McDonagh TA, Metra M, Adamo M, et al. 2021 ESC Guidelines for the diagnosis and treatment of acute and chronic heart failure: Developed by the Task Force for the diagnosis and treatment of acute and chronic heart failure of the European Society of Cardiology (ESC) With the special contribution of the Heart Failure Association (HFA) of the ESC. *European Heart Journal* 2021; **42**(36): 3599-726.
